# Correction: Enhancing the antibacterial efficacy of vancomycin analogues: targeting metallo-β-lactamases and cell wall biosynthesis

**DOI:** 10.1039/d5sc90040a

**Published:** 2025-02-17

**Authors:** Paramita Sarkar, Weipan Xu, Melissa Vázquez-Hernández, Geetika Dhanda, Shubhandra Tripathi, Debajyoti Basak, Hexin Xie, Lea Schipp, Pascal Dietze, Julia E. Bandow, Nisanth N. Nair, Jayanta Haldar

**Affiliations:** a Antimicrobial Research Laboratory, New Chemistry Unit, Jawaharlal Nehru Centre for Advanced Scientific Research Jakkur Bengaluru 560064 Karnataka India jayanta@jncasr.ac.in +91 802208 2565; b School of Pharmacy, East China University of Science and Technology 130 Meilong Rd. Shanghai 200237 China; c Applied Microbiology, Faculty of Biology and Biotechnology, Ruhr University Bochum Universitätsstraße 150 44780 Bochum Germany; d Department of Chemistry, Indian Institute of Technology Kanpur Kanpur 208016 India; e School of Advanced Materials, Jawaharlal Nehru Centre for Advanced Scientific Research Jakkur Bengaluru 560064 Karnataka India

## Abstract

Correction for ‘Enhancing the antibacterial efficacy of vancomycin analogues: targeting metallo-β-lactamases and cell wall biosynthesis’ by Paramita Sarkar *et al.*, *Chem. Sci.*, 2024, **15**, 16307–16320, https://doi.org/10.1039/D4SC03577A.

In the original version of this manuscript, the name of the author Nisanth N. Nair was incorrect and the given affiliation details for this author contained an error. This Correction contains the intended information for the author.

The Royal Society of Chemistry apologises for these errors and any consequent inconvenience to authors and readers.

